# Composition and biochemical properties of l‐carnitine fortified Makgeolli brewed by using fermented buckwheat

**DOI:** 10.1002/fsn3.803

**Published:** 2018-10-11

**Authors:** Namhyeon Park, Thi Thanh Hanh Nguyen, Gang‐Hee Lee, Shi‐Na Jin, So‐Hyung Kwak, Tae‐Kyung Lee, Yeong‐Hwan Choi, Seong‐Bo Kim, Atsuo Kimura, Doman Kim

**Affiliations:** ^1^ Graduate School of International Agricultural Technology Seoul National University Pyeongchang‐gun Gangwon‐do Korea; ^2^ Institute of Food Industrialization Institutes of Green Bio Science and Technology Center for Food and Bioconvergence Seoul National University Pyeongchang‐gun Gangwon‐do Korea; ^3^ Kooksoondang Brewery Co., LTD. Hoengseong‐gun Gangwon‐do Korea; ^4^ CJ CheilJedang, Life Ingredient and Material Research Institute Suwon South Korea; ^5^ Research Faculty of Agriculture Hokkaido University Sapporo Japan

**Keywords:** antioxidant activity, buckwheat, l‐carnitine, quercetin, *Rhizopus oligosporus*, rutin

## Abstract

Makgeolli is a traditional Korean alcoholic rice beverage. It is brewed of ingredients containing starch, Nuruk, and water. In order to improve the quality and functionality of Makgeolli, the *Rhizopus oligosporus* fermented buckwheat containing 18.7 mg/kg of l‐carnitine were utilized to brew l‐carnitine fortified Makgeolli with rice. Makgeolli was prepared in two‐stage fermentation method and total rutin and quercetin in each fermented buckwheat Makgeolli were increased 1.8‐fold greater than buckwheat Makgeolli. DPPH antioxidant activity was enhanced in fermented buckwheat Makgeolli than buckwheat Makgeolli (21.9%–65.7%). The amounts of l‐carnitine in rice Makgeolli, buckwheat Makgeolli, and fermented buckwheat Makgeolli were 0.9, 0.8–1.0, and 1.0–1.9 mg/L, respectively. The fermented buckwheat Makgeolli not only promoted health benefit by increasing l‐carnitine and flavonols, but also made effective alcohol production (2.8%–8.4%) compared to common buckwheat Makgeolli, indicating the potential industrial application with health benefits.

## 1 INTRODUCTION

Makgeolli is a traditional Korean alcoholic beverage. It is brewed by fermentation of ingredients containing starch, Nuruk (a traditional fermentation starter composing of various microorganisms such as fungi, yeasts, and lactic acid bacteria), and water (Baek et al., 2010). Makgeolli contains vitamin B complex, organic acids, and bioactive substances with yeasts, resulting in high nutritional and functional values, including antioxidant property (Kim, Park, & Sung, 2012). The size and type of microorganisms in Nuruk are continuously changed at different stages of Makgeolli fermentation. They significantly remain in the final product (Kim et al., 2015). These microorganisms not only participate in saccharification and alcoholic fermentation (Nile, 2015), but also contribute to Makgeolli's unique white creamy texture and flavor (Lee & Choi, 2005). However, because of a lack of unique characteristics, inferior acceptability, and functionality, popularity of Makgeolli has been declining (Kim, Chang, Ko, & Jeong, 2013). To improve the quality of Makgeolli with microbial activities and functional characteristics, utilization of raw materials and manufacture processes have been studied (Kim, Lee, Lee, Choi, & Lee, 2004; Kim, Chang, et al., 2013).

Buckwheat (*Fagopyrum spp*.) is a regional specialty in Bongpyeong of Korea. It is a good source of nutritionally valuable protein, lipid, dietary fiber, minerals, flavonoids, fagopyrin, tocopherols, and phenolic substances such as 3‐flavanols, rutin, phenolic acids, and their derivatives (Holasova et al., 2002; Jiang et al., 2007; Oomah, Campbell, & Mazza, 1996). Among buckwheat species, common buckwheat (*Fagopyrum esculentum*) and Tartary buckwheat (*Fagopyrum tataricum*) are cultivated as human food sources. Common buckwheat has a sweet taste with a large seed size whereas Tartary buckwheat has a bitter taste with a small seed size. Tartary buckwheat seeds contain 100‐fold higher amounts of rutin compared to common buckwheat seeds (Fabjan et al., 2003; Jiang et al., 2007). Common buckwheat fermentation by using *Rhizopus oligosporus* has been reported in our previous study (Park et al., 2017). During buckwheat fermentation by *R. oligosporus*, macromolecules are hydrolyzed by enzymes and corresponding hydrolytic products are coupled with metabolism which can change biochemical compositions of food substrates (Handoyo & Morita, 2006; de Reu, Linssen, Rombouts, & Nout, 1997). l‐carnitine can be synthesized from lysine and methionine (Bremer, 1983). Buckwheat was chosen due to its higher content of precursors lysine and methionine for l‐carnitine synthesis when compared with those of other crops (Park et al., 2017). Buckwheat without additional nutrients has been fermented using *R. oligosporus*, producing four times higher amount of l‐carnitine than original buckwheat (Park et al., 2017). l‐carnitine is a quaternary ammonium compound naturally found in meat (Walter & Schaffhauser, 2000). Its major role is a carrier of long‐chain fatty acid into mitochondria for beta‐oxidation. It has good influence on ischemic heart disease and recovery after exercise (Walter & Schaffhauser, 2000).

Makgeolli using buckwheat has been studied (Cho, Seo, Lee, & Cho, 2012; Kang, Choi, Choi, Yeo, & Jeong, 2014). Cho et al. (2012) have exploited several cereals, including buckwheat, to brew Makgeolli and compare differences of pH and alcohol in final Makgeolli. In addition, Kang et al. (2014) have analyzed various commercial Makgeolli, including buckwheat Makgeolli. However, these previous researches only indicated general characteristics of buckwheat Makgeolli such as pH, acidity, alcohol, reducing sugar, aminoacidicity, color, and volatile acidity (Cho et al., 2012; Kang et al., 2014). Therefore, in this study, we focus on using combination of common and Tartary buckwheat or mix of common and Tartary fermented buckwheat by *R. oligosporus* as ingredients to brew functionally‐enhanced Makgeolli. Their chemical composition, antioxidant properties, and general qualities (pH, alcohol, and acidity) were then investigated. Especially, functional compounds (l‐carnitine, rutin, quercetin) in these Makgeolli were analyzed using LC/MS.

## 2 MATERIALS AND METHODS

### 2.1 Preparation of fermented buckwheat

*Rhizopus oligosporus* was identified using Biolog (Biolog Inc., CA, USA) and 16S rRNA as described in our previous report (Park et al., 2017). *R. oligosporus* was incubated on Potato Dextrose Agar (Difco, Detroit, MI, USA) plates at 28°C for sporulation. Fermentation of buckwheat was carried out as described in our previous report (Park et al., 2017) with some modifications. Briefly, 250 g of common buckwheat seed (Bongpyeong, Korea) were mixed with 250 g of Tartary buckwheat seed (Bongpyeong) and soaked in distilled water for 12 hr. After draining, soaked buckwheat seeds were autoclaved at 121°C for 15 min. These autoclaved buckwheat seeds were then cooled to ambient temperature (20–22°C) and moved into a tetragonal stainless‐steel container and inoculated with 5% (v/w) *R. oligosporus* (1.6 × 10^6^ spores/ml). After covering the container with wrapping paper, buckwheat seeds were incubated at 28°C for 3 days. These fermented buckwheat seeds were then lyophilized (Eyela FD‐550; Rikakikai Co., Tokyo, Japan) at 0°C under 10 Pa for 5 days and stored at −20°C for further study. Common buckwheat, Tartary buckwheat, and mixture of common buckwheat and Tartary buckwheat seed (1:1) without fermentation as control were prepared as described above.

### 2.2 Brewing of l‐carnitine enhanced Makgeolli with fermented buckwheat seed

Forty grams of rice (Nonghyup, Naju, Korea) were soaked for 12 hr and autoclaved at 121°C for 15 min (Jinju Gokja, Seoul, Korea). Steamed rice was then completely cooled and moved into a plastic bottle with 8 g of Nuruk and 80 ml of distilled water. The mixture was incubated at 22°C and stirred twice a day to prepare seed mash. After 2 days, the same amount of steamed rice, Nuruk, and distilled water were added into the seed mash for first‐stage fermentation. During the first stage, the mixture was stirred once daily. After 1 day of fermentation, different cereals were added for second‐stage fermentation. For rice Makgeolli, twice the amount of the same material used at the previous stage was added. For buckwheat and fermented buckwheat Makgeolli, different ratios of rice to steamed buckwheat seed (10, 20, or 40 g in 80 g of total second‐stage cereals) or fermented buckwheat seed powder were added with additional Nuruk and water. These mixtures were incubated at 22°C for 5 days. The final products were roughly filtered with two folds of cotton and stored at −20°C for further analyses.

### 2.3 Analyses of ethanol, pH, and acidity

Makgeolli was centrifuged at 12,600×*g* for 10 min and the supernatant was used to determine pH (Suntex, Taipei, Taiwan). Makgeolli samples were titrated using 0.1 M NaOH solution (Lee, Haq, Saravana, Cho, & Chun, 2017). Acidity was calculated as acetic acid based on the volume of NaOH used for titration to pH 8.2. To determine alcohol content, 100 ml of Makgeolli was distilled first with a rotary evaporator (Hei‐VAP, Heidolph, Germany). The final volume was then adjusted to 100 ml. Alcohol content of Makgeolli was then measured using an alcohol hydrometer (Joylab, Seoul, Korea).

### 2.4 Analysis of l‐carnitine content

For sample preparation, one gram of milled Makgeolli ingredients (rice, buckwheat seed, or fermented buckwheat seed) was added into 10 ml distilled water and extracted for 1 hr. After centrifugation at 8,000×*g* for 10 min at 4°C, 100 μl supernatant was mixed with 900 μl acetonitrile and centrifuged at 12,600×*g* for 10 min. After filtration with a 0.2 μm membrane syringe filter (Sartorius AG, Germany), 1 μl sample was injected into the LC/MS. For Makgeolli sample preparation, 100 μl sample was mixed with the same volume of distilled water and diluted with acetonitrile followed by centrifugation, filtration, and injection as described above. The used system was Waters Acquity H‐class with Waters QDa detector (Waters, MA, USA) with Waters Acquity UPLC Beh Hilic 1.7 μm, 2.1 × 100 mm column. l‐carnitine contents in different kinds of ingredients and Makgeolli were determined using a LC/MS system (Waters) as described in our previous report (Park et al., 2017). A calibration curve was prepared with an external standard method (0.01, 0.05, 0.1, 0.5, and 1 μg/ml). The linearity between concentration of standards and area was evaluated (*r* ^2^ > 0.99). Recovery was confirmed by standard addition technique (0.0125, 0.025, 0.05, and 0.1 μg/ml) in order to determine the matrix effect of ingredients and Makgeolli on quantification. Each recovery was analyzed by one‐sample *t*‐test in SPSS version 23.0 for Windows (SPSS Inc., Chicago, IL, USA) to evaluate significant difference from 100% (*p *<* *0.05; Supporting information Table S1).

### 2.5 Analyses of rutin and quercetin contents

One gram of rice, buckwheat seed, and fermented buckwheat seed were extracted with 10 ml of 70% (v/v) ethanol for 1 hr and serially diluted (10 and 100 times) by acetonitrile. Final Makgeolli samples were mixed with the same volume of distilled water and deproteinized using acetonitrile. All samples were filtered using a 0.2 μm membrane syringe filter. Then 1 μl of each sample was injected into LC/MS (Waters H‐class equipped with QDa detector, MA, USA). Kromasyl C_18_ column (1.8 μm, 2.1 × 100 mm) was used to analyze rutin and quercetin contents with solvent A (100% triple distilled water with 0.1% (v/v) formic acid) and solvent B (100% acetonitrile with 0.1% (v/v) formic acid). Conditions for the mass detector were as follows: electrospray ionization (ESI) negative, 609.5 m/z, capillary energy (CE) of 0.8 kV, cone voltage (CV) of 25 V for rutin; ESI positive, 303 m/z, CE of 1.5 kV, and CV of 10 V for quercetin. As blank, 90% (v/v) acetonitrile was used. Calibration curve linearity ranged from 0.05 to 5 μg/ml for quercetin. It ranged from 0.1 to 5 μg/ml for rutin (*r* ^2 ^> 0.99). Recovery tests for buckwheat and Makgeolli were performed by standard addition method with *t*‐test (*p *<* *0.05) to evaluate significant difference (Supporting information Table S1).

### 2.6 Analysis of reducing sugar content

Reducing sugar content in Makgeolli ingredients was determined using DNS method (Gonçalves, Rodriguez‐Jasso, Gomes, Teixeira, & Belo, 2010; Park, Kim, & Jeong, 2018) with glucose as standard. Samples were extracted by the same process as for analysis of l‐carnitine. They were measured with a different dilution rate.

### 2.7 Analysis of antioxidant activity

Antioxidant activities of Makgeolli were evaluated by 2,2‐diphenyl‐1‐picryl‐hydrazyl (DPPH) radical scavenging method (Kim et al., 2012). Briefly, 10 μl of each Makgeolli sample and water as control were mixed with 100 μM DPPH at 37°C for 30 min in total darkness. After centrifuging the mixture at 12,600×*g* for 10 min, the absorbance of each supernatant was measured using a microplate reader (Molecular Device, Sunnyvale, CA, USA) at 517 nm. DPPH radical scavenging activity (SC) was converted into percentage of antioxidant activity as follows (Choi, Kang, & Mok, 2018):

SC(%)=(Abs of control ‐ Abs of sample)/(Abs of control)×100

### 2.8 Statistical analysis

Each analysis of general and functional compounds was repeated three times. Results of test were presented as mean ± standard deviation. Analysis of variance (ANOVA) was conducted using SPSS version 23.0 for Windows (SPSS Inc.). Levene's test for equality of variances was conducted first. Significant difference was evaluated using Scheffe's method between different Makgeolli samples. Significantly different Makgeolli was indicated by different superscript lower case alphabet in tables and figures. Statistical significance was considered at *p *<* *0.05.

## 3 RESULTS AND DISCUSSION

### 3.1 Analyses of rutin, quercetin, l‐carnitine, and reducing sugar contents in Makgeolli ingredients

Amounts of rutin and quercetin in raw mixed buckwheat seed were 7.3 and 1.1 g/kg, respectively. In soaked and steamed buckwheat, their amounts were 10.8 and 0.6 g/kg, respectively. In fermented buckwheat, amounts of rutin and quercetin were 7.1 and 1 g/kg, respectively (Table 1). Rutin and quercetin were not detected in rice (Table 1). By soaking and steaming, the content of rutin in buckwheat was increased 47.9% while the content of quercetin in buckwheat was decreased 45.5%. These contents were significantly (*p *<* *0.05) different from those in raw ones. Our results were consistent with previous reports (Li, Li, Ding, & Park, 2008; Qin, Wu, Yao, & Ren, 2013), showing that rutin content was increased by steam‐treating soaked buckwheat materials. Rutin in buckwheat was hydrolyzed in a few minutes upon addition of water because of the presence of rutin‐degrading enzymes in Tartary buckwheat seed (Yasuda & Nakagawa, 1994) and flavonol 3‐glucosidase in Tartary buckwheat testa (Suzuki, Honda, Funatsuki, & Nakatsuka, 2002). However, activities of these enzymes are completely inhibited at 80–85°C (Suzuki et al., 2002; Yasuda & Nakagawa, 1994). Steaming might inhibit rutin degradation and a catalytic reverse shift reaction of rutin‐synthesizing could take place, thus increasing the extraction of flavonoids from the material (Barber & Behrman, 1991). Further study is needed to obtain clearer explanation.

**Table 1:** Functional compounds in rice, buckwheat, and fermented buckwheat

Ingredient	Rutin (g/kg)	Quercetin (g/kg)	l‐carnitine (mg/kg)	Ruducing sugars (g/kg)
Rice (no process)	ND	ND	7.0 ± 1.7^b^	5.1 ± 0.6^c^
Rice (soak & steam)	ND	ND	3.6 ± 0.4^d^	23.1 ± 1.4^b^
Buckwheat (no process)	7.3 ± 0.2^b^	1.1^a^	5.7 ± 0.8^bc^	21.3 ± 1.1^b^
Buckwheat (soak & steam)	10.8 ± 0.2^a^	0.6^c^	4.6 ± 0.6 ^cd^	22.9 ± 1.3^b^
Buckwheat (Fermentation)	7.1 ± 0.2^b^	1.0^b^	18.7 ± 0.4^a^	110.7 ± 0.7^a^

*Notes*

ND: not detected.

^a,b,c,d^Different superscripts lower‐case alphabets after values mean different groups (*p *<* *0.05).

Rutin and quercetin contents in fermented buckwheat were similar with those of raw buckwheat (Table 1). However, rutin content in fermented buckwheat was decreased by 34.3% while quercetin content was increased by 66.7% compared to those of soaked and steamed buckwheat. Various molds such as *Penicillium* and *Aspergillus* can use rutin by rutin catabolic pathway (Tranchimand, Brouant, & Iacazio, 2010). Therefore, *R. oligosporus,* which is one of molds might use this pathway or a similar pathway, thus altering rutin and quercetin contents.

l‐carnitine contents in raw rice and buckwheat seed were 7 and 5.7 mg/kg, respectively (Table 1). After soaking and steaming, l‐carnitine content was decreased to 3.6 mg/kg in rice and 4.6 mg/kg in buckwheat (Table 1). Leaching of l‐carnitine during heat treatment with water might account for the decrease in l‐carnitine (Knüttel‐Gustavsen & Harmeyer, 2011). Fermented buckwheat contained 4.1‐fold increase in the amount of l‐carnitine (18.7 mg/kg) compared to soaked and steamed buckwheat seed with significant difference. It showed a 3.3‐fold increase over the original one (*p *<* *0.05; Table 1). Our previous study has revealed that l‐carnitine content in buckwheat extract powder after fermentation using *R. oligosporus* was increased fourfold compared to that in non‐fermented buckwheat extract powder (Park et al., 2017). Thus, although whole buckwheat seed was fermented with *R. oligosporus*, fermented buckwheat seed contained similar augmentation of l‐carnitine, resulting in improved potential functionality due to enhanced l‐carnitine with abundant flavonols of buckwheat hulls.

Reducing sugar contents of rice, soaked and steamed rice, buckwheat, soaked and steamed buckwheat, and fermented buckwheat with *R. oligosporus* are shown in Table 1. Reducing sugar contents in raw rice and buckwheat seed were 5.1 and 21.3 g/kg, respectively. Soaking and steaming process increased reducing sugar contents (to 23.1 g/kg in rice and 22.9 g/kg in buckwheat seed). Reducing sugar contents in fermented buckwheat seed was 5.2‐fold higher (110.7 g/kg) than that in the original buckwheat. These increases in reducing sugars could be attributed to hydrothermal and catalytic hydrolysis by *R. oligosporus* (Nagamori & Funazukuri 2004; Sarrette, Nout, Gervais, & Rombouts, 1992) which could affect the brewing of Makgeolli. Higher reducing sugar content might be one of the reasons why ethanol content in final fermented buckwheat Makgeolli is higher than that in unfermented buckwheat Makgeolli.

### 3.2 Characterization of different kinds of Makgeolli

The pH of rice Makgeolli, 6.25% or 12.5% buckwheat Makgeolli, or fermented buckwheat Makgeolli was 4.0 without significant difference (*p *>* *0.05; Table 2). However, the pH of 25% buckwheat Makgeolli was 4.1 and that of fermented buckwheat Makgeolli was 4.2, which was significantly (*p *<* *0.05) different from that of rice Makgeolli (Table 2). Buckwheat contains more essential amino acids than rice (Mota et al., 2016) and fermentation by *R. ligosporus* further increased these amino acid contents in buckwheat (Wronkowska, Christa, Ciska, & Soral‐Śmietana, 2015). The amino acid contents of Makgeolli were shown to be associated with the raw material (Kang et al., 2014). As a result, the increased pH in 25% buckwheat Makgeolli or fermented buckwheat Makgeolli may be due to increased amino acid contents and their buffering capacity (Thomas, Hynes, & Ingledew, 2002).

**Table 2:** General characteristics of different Makgeolli

Type	pH	Alcohol (%)	Acidity (%)	Sugar (%)
RM	4.0^b^	15.6 ± 0.5^a^	0.5^d^	9.8 ± 0.8^a^
BM_6.25	4.0^b^	14.3 ± 0.6^ab^	0.5^d^	9.4 ± 0.2^ab^
BM_12.5	4.0^b^	14.5 ± 1.1^ab^	0.6 ^cd^	9.6 ± 0.4^ab^
BM_25	4.1^ab^	12.5 ± 1.1^b^	0.6^d^	8.1 ± 0.4^b^
FBM_6.25	4.0^b^	15.5 ± 0.5^a^	0.7^bc^	10.2 ± 0.7^a^
FBM_12.5	4.0^b^	14.9 ± 0.4^ab^	0.7^ab^	10.1 ± 0.8^a^
FBM_25	4.2^a^	13.2 ± 1.6^ab^	0.8^a^	10.1 ± 0.4^a^

*Notes*

BM: buckwheat Makgeolli; FBM: fermented buckwheat Makgeolli; RM: rice Makgeolli.

Makgeolli and the numbers with types of Makgeolli mean the ratio of buckwheat or fermented buckwheat in final Makgeolli.

^a,b,c^Different superscripts of lower‐case letters after values mean different groups (*p *<* *0.05).

Results of acidity of Makgeolli samples are shown in Table 2. The acidity of fermented buckwheat Makgeolli was significantly (*p *<* *0.05) higher (40%–60%) than that of rice or buckwheat Makgeolli (Table 2). The increase in acidity of fermented buckwheat Makgeolli could be explained by the characteristic of fermented buckwheat itself since it could contain organic acids produced by *Rhizopus* (Magnuson & Lasure, 2004).

Alcohol content was 15.6% for rice Makgeolli, 14.3% for 6.25% buckwheat Makgeolli, 14.5% for 12.5% buckwheat makgeolli, 12.5% for 25% buckwheat Makgeolli, 15.5% for 6.25% fermented buckwheat Makgeolli, 14.9% for 12.5% fermented buckwheat Makgeolli, and 13.2% for 25% fermented buckwheat Makgeolli (Table 2). The lower alcohol content in buckwheat or fermented buckwheat Makgeolli compared to that in rice Makgeolli might occur by the fact that buckwheat seed contains higher dietary fiber and a smaller amount of starch (Bonafaccia, Marocchini, & Kreft, 2003) which can affect total glucose amounts for yeast to use during Makgeolli brewing. Also, the reason for higher alcohol content in fermented buckwheat Makgeolli than buckwheat Makgeolli could be pre‐fermentation polysaccharidase which can be produced by *R. oligosporus* (Sarrette et al., 1992). It can degrade unavailable carbohydrate of seeds into additional available sugars. These additional glucoses by fermentation might be exploited by yeasts (which produce ethanol) later in the Makgeolli brewing process.

The primary purpose of Makgeolli brewing is to produce ethanol, an important component influencing flavor and preservation (Kim, Park, et al., 2013). From these results, pre‐fermentation of buckwheat seed seems to be useful for brewing Makgeolli, especially when whole seeds with hulls are used.

Sugar contents in different kinds of Makgeolli are shown in Table 2. Among three kinds of Makgeolli, fermented buckwheat Makgeolli has slightly higher sugar content than rice or buckwheat Makgeolli.

### 3.3 Analyses of rutin, quercetin, l‐carnitine, and antioxidant effects in different Makgeolli

**Figure 1:**
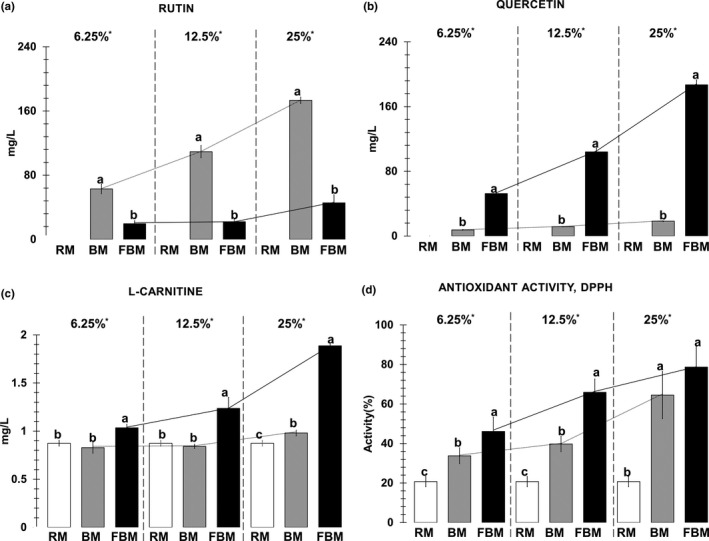
Analysis of rutin, quercetin, l‐carnitine and DPPH antioxidant activity. (a) rutin, (b) quercetin, (c) l‐carnitine, and (d) DPPH antioxidant activity in different Makgeolli samples. RM: rice Makgeolli; BM: buckwheat Makgeolli; FBM: fermented buckwheat Makgeolli. ^a,b,c^ Different lower‐case letters on bar graphs mean significant difference at each supplement level (*p *<* *0.05). * Superscript star mark indicates the ratio of supplements in BM or FBM

Amounts of rutin in 6.25%, 12.5%, and 25% buckwheat Makgeolli were 63.0, 109.3, and 173.3 mg/L, respectively. They were 19.6, 22.1, and 45.6 mg/L in 6.25%, 12.5%, and 25% fermented buckwheat Makgeolli, respectively. However, rutin was not detected in rice Makgeolli (Figure 1a). Amounts of quercetin were 7.6, 11.5, and 18.3 mg/L in 6.25%, 12.5%, and 25% buckwheat Makgeolli, respectively. They were 52.5, 104.1, and 187.0 mg/L in 6.25%, 12.5%, and 25% fermented buckwheat Makgeolli, respectively. Quercetin was not detected in rice Makgeolli (Figure 1b). The sum of rutin and quercetin in fermented buckwheat Makgeolli was increased (by 2.1% in 6.25% fermented buckwheat added, 4.5% in 12.5% fermented buckwheat added, and 21.4% in 25% fermented buckwheat added) compared to that in non‐fermented buckwheat Makgeolli. l‐carnitine was detected in rice Makgeolli (0.7 mg/L). There was no significant difference in l‐carnitine content between buckwheat Makgeolli samples (6.25% and 12.5%) except 25% of buckwheat Makgeolli (*p* < 0.05) (Figure 1c). Amounts of l‐carnitine were increased significantly (*p *<* *0.05) in all fermented buckwheat Makgeolli compared to those in rice Makgeolli and buckwheat Makgeolli with same supplement ratio. In addition, the calculated conversion ratio of l‐carnitine from ingredients to final Makgeolli was almost stable for all Makgeolli samples (51.4% ± 3.1). This result indicates that l‐carnitine in final Makgeolli is dependent on l‐carnitine levels in Makgeolli ingredients. To the best of our knowledge, l‐carnitine content in Makgeolli and l‐carnitine fortified Makgeolli are reported for the first time in this paper.

All buckwheat and fermented buckwheat Makgeolli showed significantly increased antioxidant activities compared to rice Makgeolli (*p *<* *0.05; Figure 1d). Antioxidant activity in individual fermented buckwheat Makgeolli was higher than that of each buckwheat Makgeolli using the same percent of supplement. Antioxidant activities of 6.25% and 12.5% of fermented buckwheat Makgeolli were significantly different from those of 6.25% and 12.5% of buckwheat Makgeolli (*p *<* *0.05; Figure 1d). The higher antioxidant activity of buckwheat and fermented buckwheat Makgeolli compared to rice Makgeolli might be due to the presence of l‐carnitine, tocopherols, phenolic substance such as 3‐flavanols, rutin, phenolic acids, and their derivatives in buckwheat which are known to possess antioxidant activity (Holasova et al., 2002; Oomah et al., 1996). Especially, the higher antioxidant activity of fermented buckwheat Makgeolli than that of buckwheat Makgeolli might result from *R. oligosporus* that can produce α‐amylase and endogenous carbohydrate‐cleaving enzymes, since these enzymes can produce polyphenols from carbohydrates‐conjugated phenolic compounds during fermentation of buckwheat (McCue & Shetty, 2003). In addition, *R. oligosporus* is known to produce β‐glucosidase, β‐glucuronidase, or xylanase to degrade cell wall matrix (Huynh, Van Camp, Smagghe, & Raes, 2014). Thus, during fermentation, *R. oligosporus* might bio‐convert bound‐phenolic compounds into unbound phenolics as aglycone forms (Huynh et al., 2014). Furthermore, during the process of brewing Makgeooli, different kinds of fungi (*Aspergillus*,* Rhizopus*), yeast, and various lactic acid bacteria (Nile, 2015) can produce tannase, phenolic acid decarboxylase, benzyl alcohol dehydrogenase, and β‐glucosidase that can degrade some phenolic compounds (Landete et al., 2010). Therefore, fermentation processes might release phenolic compounds from plant matrixes followed by metabolic pathways of flavonoids, including glycosylation, deglycosylation, ring cleavage, methylation, glucuronidation, and sulfate conjunction in ways to produce new bioactive compounds (Huynh et al., 2014). Increased contents of flavonoids might influence on DPPH‐radical SC, resulting in higher antioxidant activity of fermented buckwheat Makgeolli than buckwheat Makgeolli as well as rice Makgeolli. Quercetin has higher antioxidant activity compared to rutin (SC_50_ of quercetin = 37.4 μM, SC_50_ of rutin ≥1,000 μM) (Kong, Mat‐Junit, Aminudin, Ismail, & Abdul‐Aziz, 2012; Nguyen et al., 2015). In addition, an increase of l‐carnitine contained in fermented buckwheat Makgeolli could increase the antioxidant activity because l‐carnitine is an antioxidant compound that can prevent oxidative stress and regulate cellular respiration by nitric oxide (Brown, 1999). These results suggest that pre‐fermentation technique of buckwheat seed by *R. oligosporus* prior to brewing Makgeolli could increase antioxidant activities of final product with enhanced functional compounds (l‐carnitine, rutin, and quercetin).

## 4 CONCLUSION

In this study, for the first time, l‐carnitine fortified Makgeolli was successfully brewed using rice and fermented whole buckwheat seed prepared with *R. oligosporus*. Fermented buckwheat seed contained increased l‐carnitine content and maintained its richness in rutin and quercetin as major functional compounds. This result indicates that it is possible to enhance the functionality and widen the application of buckwheat as a food ingredient. In addition, l‐carnitine fortified Makgeolli using fermented buckwheat seed with natural fortification of l‐carnitine and flavonols is expected to enhance health effects. As a result, this brewing technique with pre‐fermentation seems to be effective when whole buckwheat seeds are used because it enhances functional compounds with alcohol production and antioxidant activity compared to original buckwheat Makgeolli.

## CONFLICT OF INTEREST

None declared.

## ETHICAL STATEMENT

All authors were actively involved in the work leading to the manuscript and will hold themselves jointly and individually responsible for its content. This study does not involve any human or animal testing.
