# Supplementary material for: Composition and biochemical properties of l‐carnitine fortified Makgeolli brewed by using fermented buckwheat
Source: Food Sci Nutr. 2018 Oct 11;6(8):2293–300. doi: 10.1002/fsn3.803 (PMC6261218; doi:10.1002/fsn3.803)
Supplement: Supplementary file 1 [file FSN3-6-2293-s001.docx]

**Supplementary material**

**Composition and Biochemical Properties of L-Carnitine Fortified Makgeolli Brewed by Using Fermented Buckwheat**

**Namhyeon Park,^1^ Thi** **Thanh** **Hanh** **Nguyen,^2^ So-Hyung Kwak,^1^** **Shi-Na Jin,^1^ Tae-Kyung Lee,^1^ Gang-Hee Lee,^1^ Yeong-Hwan Choi,^3^ Seong-Bo Kim,^4^ Atsuo Kimura,^5^ and Doman Kim^1, 2*^**

^1^Graduate School of International Agricultural Technology, Seoul National University, Pyeongchang-gun, Gangwon-do, 25354, Korea

^2^Institute of Food Industrialization, Institutes of Green Bio Science & Technology, and Center for Food and Bioconvergence, Seoul National University, Pyeongchang-gun, Gangwon-do, 25354, Korea

^3^Kooksoondang Brewery Co., LTD., **975, Gangbyeon-ro, Hyeoncheon-ri, Dunnae-myeon, Hoengseong-gun, Gangwon-do**

^4^CJ CheilJedang, Life Ingredient & Material Research Institute, Suwon, 16495, South Korea.

^5^Research Faculty of Agriculture, Hokkaido University, Sapporo 060-8589, Japan

**^*^**Corresponding author: Phone: +82-33-339-5720, Fax: +82-33-339-5716, Email: kimdm@snu.ac.kr

**Recovery of L-carnitine, rutin, and quercetin in Makgeolli**

Recovery of rutin and quercetin in buckwheat, fermented buckwheat, and all Makgeolli samples ranged from 93.7 – 103.5%. These values were not significantly (*p* > 0.05) different from 100% (Table S1). L-carnitine recovery in rice was 98.1%, which was not significantly (*p* > 0.05) different from 100% as detected in our previous study (Park et al. 2017) about the recovery of L-carnitine in buckwheat and fermented buckwheat (Table S1). However, recoveries of L-carnitine in rice, buckwheat, and fermented buckwheat Makgeolli were 80.1, 77.0, and 70.1%, respectively. These values were significantly (*p* < 0.05) different from 100% (Table S1). These recovery values were applied to L-carnitine contents in final Makgeolli. Many unknown factors might lower the recovery of L-carnitine in Makgeolli during analyses. Makgeolli had high viscosity with water soluble high solid contents owing to rough filtration of Makgeolli preparation and relatively high amount of pellet was occurred during deproteinization. Co-precipitation of L-carnitine might be one of such reasons. This characteristic could affect the recovery of L-carnitine in Makgeolli (Table S1).

**Table S1.** Recovery analyses of the L-carnitine, rutin, and quercetin in samples by LC/MS.

| Compounds | Type | Sample | Recovery (%)^a^ |
| --- | --- | --- | --- |
| L-carnitine | Ingredients | Rice | 98.1 ± 15.9 |
|  |  | Buckwheat | 101.1 ± 9.3 ^1^ |
|  |  | Fermented buckwheat | 100.6 ± 4.5 ^1^ |
|  | Products | Rice Makgeolli | 80.1 ± 9.8* |
|  |  | Buckwheat Makgeolli | 77.0 ± 6.4* |
|  |  | Fermented buckwheat Makgeolli | 70.1 ± 9.8* |
| Rutin | Ingredient | Buckwheat | 103.5 ± 6.0 |
|  |  | Fermented buckwheat | 96.3 ± 10.9 |
|  | Products | Buckwheat Makgeolli | 95.8 ± 7.8 |
|  |  | Fermented buckwheat Makgeolli | 92.7 ± 6.9 |
| Quercetin | Ingredient | Buckwheat | 96.6 ± 2.6 |
|  |  | Fermented buckwheat | 95.5 ± 2.5 |
|  | Products | Buckwheat Makgeolli | 93.7 ± 6.5 |
|  |  | Fermented buckwheat Makgeolli | 95.0 ± 4.5 |

^a^ Recovery was calculated by three addition level in each type of samples and indicated by mean ± standard deviation (n≥3). *means recovery value is significantly different from 100% by one sample t-test (p<0.05).
